# PD04 - The environmental risk factors and prevalence of childhood allergic diseases in an industrial city

**DOI:** 10.1186/2045-7022-4-S1-P4

**Published:** 2014-02-28

**Authors:** Chang Sun Sim, In-Bo Oh, Cheol-In Yoo, Yangho Kim, Jiho Lee

**Affiliations:** 1 Ulsan University Hospital, University of Ulsan College of Medicine, Ulsan, Korea Republic

## 

This study aims to investigate the allergic disease (AD) prevalence for elementary school children in an industrial city of Ulsan and identify major environmental risk factors associated with AD prevalence.

Data on the physician-diagnosed prevalence in the past 1 year and potential risk factors of AD (asthma, allergic rhinitis and atopic dermatitis) were collected by a questionnaire including ISAAC questions from the 2009-2010 survey of 4,067 children living in different urban environments. Exposure to outdoor air pollution was estimated by using annual mean concentrations of pollutants (PM10, O_3_, NO_2_, SO_2_ and CO) obtained from monitoring sites near the participant’s residence.

Our survey results showed that the AD prevalence rate ranged between 26.2% and 35.9%. Children living in polluted areas (near industrial and central urban areas) had about 10% higher prevalence of AD than those living in coastal or suburban areas. The chi-square test demonstrated that this local difference was statistically significant before and after adjustment of major confounder such as parental AD history and education. The results of the logistic regression analysis showed that AD prevalence was significantly associated independently with socio-economic indices and indoor/outdoor environmental factors. The multivariate analysis indicated that statistically significant and robust association between several environmental factors (ventilation status, exposure to diesel exhaust, and outdoor PM10/O_3_ pollution) and the prevalence of AD was found after adjustment by confounders. The adjusted odd ratios for the AD prevalence were 1.24 (95% CI: 1.03-1.49) and 1.79 (95% CI: 1.17-2.75) with increase in PM10 level of 10 ㎍m^-3^ and O_3_ level of 10 ppb, respectively.

Although there should be other risk factors for AD, our results suggest that living in polluted area and exposure to high levels of air pollutants can contribute to the increased risk of childhood AD.

